# Development and validation of machine learning models to predict MDRO colonization or infection on ICU admission by using electronic health record data

**DOI:** 10.1186/s13756-024-01428-y

**Published:** 2024-07-06

**Authors:** Yun Li, Yuan Cao, Min Wang, Lu Wang, Yiqi Wu, Yuan Fang, Yan Zhao, Yong Fan, Xiaoli Liu, Hong Liang, Mengmeng Yang, Rui Yuan, Feihu Zhou, Zhengbo Zhang, Hongjun Kang

**Affiliations:** 1 Medical School of Chinese PLA, Beijing, 100853 China; 2 Department of Critical Care Medicine, The First Medical Centre, Chinese PLA General Hospital, No. 28, Fuxing Road, Haidian District, Beijing, 100853 China; 3 Center for Artificial Intelligence in Medicine, Chinese PLA General Hospital, No. 28, Fuxing Road, Haidian District, Beijing, 100853 China

**Keywords:** Multidrug-resistant organisms, Machine learning, Predictive modeling, Intensive care unit, Infection

## Abstract

**Background:**

Multidrug-resistant organisms (MDRO) pose a significant threat to public health. Intensive Care Units (ICU), characterized by the extensive use of antimicrobial agents and a high prevalence of bacterial resistance, are hotspots for MDRO proliferation. Timely identification of patients at high risk for MDRO can aid in curbing transmission, enhancing patient outcomes, and maintaining the cleanliness of the ICU environment. This study focused on developing a machine learning (ML) model to identify patients at risk of MDRO during the initial phase of their ICU stay.

**Methods:**

Utilizing patient data from the First Medical Center of the People’s Liberation Army General Hospital (PLAGH-ICU) and the Medical Information Mart for Intensive Care (MIMIC-IV), the study analyzed variables within 24 h of ICU admission. Machine learning algorithms were applied to these datasets, emphasizing the early detection of MDRO colonization or infection. Model efficacy was evaluated by the area under the receiver operating characteristics curve (AUROC), alongside internal and external validation sets.

**Results:**

The study evaluated 3,536 patients in PLAGH-ICU and 34,923 in MIMIC-IV, revealing MDRO prevalence of 11.96% and 8.81%, respectively. Significant differences in ICU and hospital stays, along with mortality rates, were observed between MDRO positive and negative patients. In the temporal validation, the PLAGH-ICU model achieved an AUROC of 0.786 [0.748, 0.825], while the MIMIC-IV model reached 0.744 [0.723, 0.766]. External validation demonstrated reduced model performance across different datasets. Key predictors included biochemical markers and the duration of pre-ICU hospital stay.

**Conclusions:**

The ML models developed in this study demonstrated their capability in early identification of MDRO risks in ICU patients. Continuous refinement and validation in varied clinical contexts remain essential for future applications.

**Supplementary Information:**

The online version contains supplementary material available at 10.1186/s13756-024-01428-y.

## Background

Antimicrobial resistance constitutes a major threat to public health [1]. Bacteria that are resistant to three or more classes of antimicrobial agents are typically categorized as multidrug-resistant organisms (MDRO). International experts collaboratively established an interim standard for defining MDRO in 2012, targeting five prevalent bacterial species: *Staphylococcus aureus*, *Enterococcus spp.*, *Enterobacteriaceae*, *Pseudomonas aeruginosa*, and *Acinetobacter spp.*, and meticulously specified the antimicrobial categories for defining multidrug resistance in these bacteria [2]. The proliferation of MDRO infections contributes to a rise in the misuse of antimicrobials, heightens the likelihood of adverse drug events, extends the duration of hospitalization, and increases the mortality rates among patients [3]. Intensive Care Units (ICU), characterized by extensive antimicrobial use and high bacterial resistance rates, are prominent areas for the prevalence of MDRO infections [4, 5].

Promptly identifying patients at elevated risk for MDRO colonization or infection is beneficial for curtailing the dissemination of MDRO and bettering the patients’ prognosis [6]. During the early phase of ICU admission, it is common for healthcare providers to test body fluid samples to establish an infection diagnosis. Nevertheless, the commonly employed techniques for microbial culture and drug sensitivity testing in hospitals around the globe are protracted, with the process from sample delivery to report retrieval usually spanning several days [7]. Methods proposed by Gupta et al. [8] to curb MDRO transmission and infection involve increasing laboratory test accuracy and the active cultivation of specimens from patients with potential infections. However, this strategy requires substantial medical resources [9], and during the Coronavirus Disease 2019 (COVID-19) pandemic, there were reports of hospitals interrupting MDRO screening and monitoring due to shortages in manpower and financial resources [10]. Hence, analyzing from a medical resource optimization standpoint, focused surveillance supersedes broad-based monitoring. The development of an MDRO alert system, employing particular technological methods to intensively monitor high-risk patients, is crucial for diminishing the development and transmission of resistant bacteria and for better resource allocation in healthcare.

Machine learning (ML) has become increasingly prevalent in disease prediction models, demonstrating notable success in performance. Compared with logistic regression, ML can effectively deal with complex linear and nonlinear relationships between variables in a data set, which can greatly improve the prediction performance of diseases [11]. There are also very few studies that have cross-validated multidrug-resistant bacteria prediction models from different countries. Therefore, in this research, we propose developing a predictive model based on ML, utilizing data obtained early during a patient’s ICU stay. This model is intended to early detect patients with colonization or infection by MDRO, thereby decreasing MDRO proliferation and, to a certain degree, supporting empirical pharmacotherapy.

## Methods

### Study population and definitions

This study encompasses two datasets, one derived from the ICU of the First Medical Center of the People’s Liberation Army General Hospital (PLAGH-ICU) with patient data spanning from January 2008 to January 2019, and the other from the Medical Information Mart for Intensive Care (MIMIC-IV version 2.2) database. The MIMIC-IV database provides comprehensive clinical information on patients admitted to the ICU at Beth Israel Deaconess Medical Center in the United States between 2008 and 2019 [12]. Permission to use the data was obtained for MIMIC-IV databases (No.49,639,059). Given the de-indentified nature of the data, informed consent was waived. The datasets included information of patients who underwent microbial culture within 24 h of ICU admission. Patients under the age of 18 or those with an ICU stay shorter than 24 h were excluded. Patients detected with MDRO within 14 days prior to ICU admission were excluded (as these patients typically receive heightened clinical attention), and patients who reported a positive MDRO within 1 day of ICU admission were also excluded (eFigure 1). The data from 2008 to 2016 were used for model training, and the data from 2017 to 2019 for model validation, henceforth referred to as the training and temporal validation sets [13, 14], respectively. Due to the anonymization process in the MIMIC-IV database, which limits the exact admission year to a three-year interval, data that could not be distinctly classified as pre- or post-2017 were not included in the training or temporal validation sets.

In accordance with international expert recommendations, bacteria resistant to three or more classes of antimicrobials were labeled as MDRO, primarily encompassing multiple drug-resistant strains of *Staphylococcus aureus*, *Enterococcus spp.*, *Enterobacteriaceae*, *Pseudomonas aeruginosa*, and *Acinetobacter spp.* [2]. Furthermore, per these recommendations, *methicillin resistant staphylococcus aureus* (MRSA) was directly categorized as an MDRO. In the definition of multidrug resistance, inherent natural resistance to a particular antimicrobial agent was not considered in determining resistance status for that agent.

### Data extraction

Data were extracted for variables accessible within a 24-hour window preceding and succeeding patient admission to the ICU. These variables encompassed: (a) patient demographic data; (b) comorbidity profiles; (c) the latest laboratory test outcomes and vital sign measurements recorded immediately before and after ICU entry; (d) duration of hospitalization prior to ICU admission; (e) total count of hospital and ICU admissions; (f) duration of antimicrobial and immunosuppressant medication usage preceding ICU admission; and (g) any instances of MDRO detection within a 90-day timeframe. The MIMIC-IV database, providing extensive patient medical histories unavailable in PLAGH-ICU, was utilized for this specific data extraction.

Specimens gathered within the initial 48 h of ICU admission were tested for MDRO colonization or infection. Key outcomes like duration of ICU and hospital stays, and in-hospital mortality, were also recorded. We excluded variables with missing data exceeding 30%, and cases with over 20% missing lab test values [15, 16]. In the PLAGH-ICU and MIMIC-IV original datasets, missing data were addressed using Multivariate Imputation by Chained Equations (MICE) [17]. Following the completion of MICE imputation, each original dataset yielded five complete datasets, from which we selected one for modeling and validation.

### Model development and validation

Feature selection and model training were independently executed within the PLAGH-ICU and MIMIC-IV datasets. The process commenced with Spearman’s rank correlation for stratified clustering, isolating features without significant collinearity [18]. When two variables were found to be collinear, we typically retained one of them based on clinical relevance and input from clinical experts. These were designated as candidate features. A Random Forest algorithm then fitted a model incorporating all candidates, and permutation feature importance ranking [19] was employed to distill features for final model input. Considering the reduction of model performance loss and ease of use in clinical settings, we ultimately included the top 25 features for prediction. Diverse algorithms, including Logistic Regression (LR) [20], K-Nearest Neighbor (KNN) [21], Support Vector Classifier (SVC) [22], Random Forest (RF) [23], eXtreme Gradient Boosting (XGBoost) [24], and Multilayer Perceptron (MLP) [25], were utilized for model construction. Before training the LR, KNN, SVC, and MLP models, the dataset underwent min-max normalization.

For hyperparameter optimization, Bayesian optimization [26] in conjunction with a 5-fold cross-validation approach was employed within the training set. Post hyperparameter tuning, models were trained using training set data, followed by performance evaluation in the temporal validation set. A stacking methodology [27] was utilized to amalgamate the four most efficacious models, creating a robust ensemble model, which underwent further validation. In addition, to assess the robustness of the imputation and its potential impact on the results, we performed a sensitivity analysis by applying the developed model to the temporal validation set after removing cases with missing data.

In the final phase, leveraging variables common to both PLAGH-ICU and MIMIC-IV databases, models were re-trained using the 15 most common features and fine-tuned in one database and subjected to external validation in the other. All predictive model development processes in this study were compliant with the Transparent Reporting of a Multivariable Prediction Model for Individual Prognosis or Diagnosis (TRIPOD) principles [13].

### Statistical analysis

Continuous variables with deviations from a normal distribution in the baseline characteristics were quantified using the median and interquartile ranges to illustrate the central trend and distribution of the data. Categorical variables were presented as counts and percentages. Model classification efficacy was appraised by constructing receiver operating characteristic curves (ROC) and computing the area under these curves (AUROC). Decision curve analysis [28] and probability calibration curves [29] provided additional performance insights. The Shapley Additive Explanations (SHAP) method [30] was employed to ascertain the impact of variables on model output. The Kruskal-Wallis test was utilized for assessing differences in non-normally distributed or heteroscedastic datasets, while chi-square tests were used for rate or proportion comparisons, considering p-values below 0.05 as indicative of statistical significance. Python (3.9.16) and R (4.2.3) were the tools for machine learning modeling and statistical analyses. Python was primarily utilized for data preprocessing, feature engineering, and construction and evaluation of machine learning models (scikit-learn, pandas, numpy and shap). R is mainly used for statistical analysis, visualization, and partial data processing (dplyr, ggplot2 and pROC).

## Results

### Baseline characteristics

The PLAGH-ICU dataset encompassed 3,536 patients, with those admitted between 2008 and 2016 (2388, 67.5%) forming the training set and the rest (1148, 32.5%) allocated for validation (eFigure 1). The training set of PLAGH-ICU dataset contained 277 (11.6%) MDRO positive cases and the temporal validation set included 146 (12.72%) MDRO positive cases. MDRO positives represented 11.96% of this cohort. In MIMIC-IV, of the 3,4923 patients included, of which 23,506 (67.31%) were in the training set, 8,145 (23.32%) in validation, and 3,272 (9.37%) excluded due to unclear admission dates. The training set of MIMIC-IV dataset contained 2,299 (9.78%) MDRO positive cases and the temporal validation set included 489 (6.0%) MDRO positive cases. The MDRO colonization or infection rate was 8.81%. Tables 1 and 2 detail patient baseline characteristics, showing significant differences in ICU stay, hospital stay, and mortality rates between MDRO positive and negative patients (*p* < 0.001). Additional baseline characteristics, including vital signs and laboratory test values, are available in the supplementary materials (eTable 1 and eTable 2). MDRO rates in PLAGH-ICU were highest for *Acinetobacter spp.* (90.07%), then *Staphylococcus aureus* (73.13%), and *Enterobacteriaceae* (61.58%), with *Enterococcus spp.* and *Pseudomonas aeruginosa* at 40.55% and 34.12% (eFigure 2 A). MIMIC-IV showed *Enterobacteriaceae, Enterococcus spp.*, and *Pseudomonas aeruginosa* rates at 33.80%, 34.39%, and 29.04%, respectively, and *Staphylococcus aureus* at 29.25% (eFigure 2B). The availability of variables in both databases are demonstrated in eTable 3.

**Table 1:** Baseline characteristics of PLAGH-ICU patients

Variable	Non-MDRO	MDRO	*P*-Value
	(*n* = 3,113)	(*n* = 423)	
Age, Median [Q1, Q3]	62.2 [48.4,72.8]	60.4 [45.3,72.1]	0.113
Male, n (%)	1,980 (63.6)	301 (71.2)	0.003
BMI, median [Q1,Q3]	24.0 [21.5,26.6]	23.4 [20.2,26.1]	0.001
Admission Type, n (%)			< 0.001
emergency	1,478 (47.5)	272 (64.3)	
outpatient	1,635 (52.5)	151 (35.7)	
Number of Admissions, Median [Q1, Q3]	1.0 [1.0,1.0]	1.0 [1.0,1.0]	0.716
Number of ICU Stays, Median [Q1, Q3]	1.0 [1.0,1.0]	1.0 [1.0,1.0]	< 0.001
Days in Hospital Before ICU Admission, Median [Q1, Q3]	5.8 [0.7,10.2]	3.2 [0.1,10.8]	< 0.001
MDRO Detected Within 90 Days, n (%)	46 (1.5)	36 (8.5)	< 0.001
Comorbidities, n (%)			
Heart Failure	104 (3.3)	37 (8.7)	< 0.001
Cerebrovascular Disease	175 (5.6)	29 (6.9)	0.363
Chronic Pulmonary Disease	183 (5.9)	33 (7.8)	0.150
Liver Disease	399 (12.8)	58 (13.7)	0.662
Renal Disease	530 (17.0)	82 (19.4)	0.256
Diabetes	480 (15.4)	68 (16.1)	0.781
Shock Index, Median [Q1, Q3]	0.6 [0.5,0.8]	0.7 [0.6,0.9]	< 0.001
Major Outcomes			
Length of Hospital Stay (days), Median [Q1, Q3]	19.8 [13.7,29.8]	26.7 [14.8,47.7]	< 0.001
Length of ICU Stay (days), Median [Q1, Q3]	4.0 [2.4,8.1]	8.5 [3.9,19.6]	< 0.001
In-Hospital Death, n (%)	325 (10.4)	89 (21.0)	< 0.001

**Table 2:** Baseline characteristics of MIMIC-IV patients

Variable	Non-MDRO	MDRO	*P*-Value
	(*n* = 28,863)	(*n* = 2,788)	
Age, Median [Q1, Q3]	66.7 [54.8,78.2]	70.6 [58.8,81.7]	< 0.001
Male, n (%)	12,672 (43.9)	1,348 (48.4)	< 0.001
Weight (Median [Q1, Q3])	77.8 [65.0,93.0]	75.8 [62.8,91.6]	< 0.001
Admission Type, n (%)			
Admitted from Emergency Room	15,931 (55.2)	1,811 (65.0)	< 0.001
Transferred from Another Hospital	6,624 (22.9)	518 (18.6)	< 0.001
Transferred from Skilled Nursing Facility	405 (1.4)	99 (3.6)	< 0.001
Other	5,903 (20.5)	360 (12.9)	
Number of Hospital Admissions, median [Q1,Q3]	1.0 [1.0,1.0]	1.0 [1.0,2.0]	< 0.001
Number of ICU Stays, Median [Q1, Q3]	1.0 [1.0,2.0]	1.0 [1.0,2.0]	< 0.001
Days in Hospital Before ICU Admission, Median [Q1, Q3]	0.1 [0.0,0.4]	0.1 [0.0,0.2]	0.058
MDRO Detected Within 90 Days Prior to ICU Admission, n (%)	702 (2.4)	342 (12.3)	< 0.001
Comorbidities, n (%)			
Heart Failure	8,121 (28.1)	1,011 (36.3)	< 0.001
Cerebrovascular Disease	4,536 (15.7)	373 (13.4)	0.001
Chronic Pulmonary Disease	7,584 (26.3)	934 (33.5)	< 0.001
Liver Disease	4,074 (14.1)	477 (17.1)	< 0.001
Renal Disease	6,387 (22.1)	788 (28.3)	< 0.001
Diabetes	8,721 (30.2)	1054 (37.8)	< 0.001
Charlson Comorbidity Index, median [Q1,Q3]	5.0 [3.0,7.0]	6.0 [4.0,8.0]	< 0.001
Shock Index, Median [Q1, Q3]	0.7 [0.6,0.9]	0.8 [0.6,1.0]	< 0.001
Major Outcomes			
Length of Hospital Stay (days), Median [Q1, Q3]	7.5 [4.6,13.4]	8.7 [5.1,15.0]	< 0.001
Length of ICU Stay (days), Median [Q1, Q3]	2.5 [1.6,4.6]	2.9 [1.8,5.6]	< 0.001
In-Hospital Death, n (%)	3,688 (12.8)	497 (17.8)	< 0.001

### Model evaluation

**Fig. 1:**
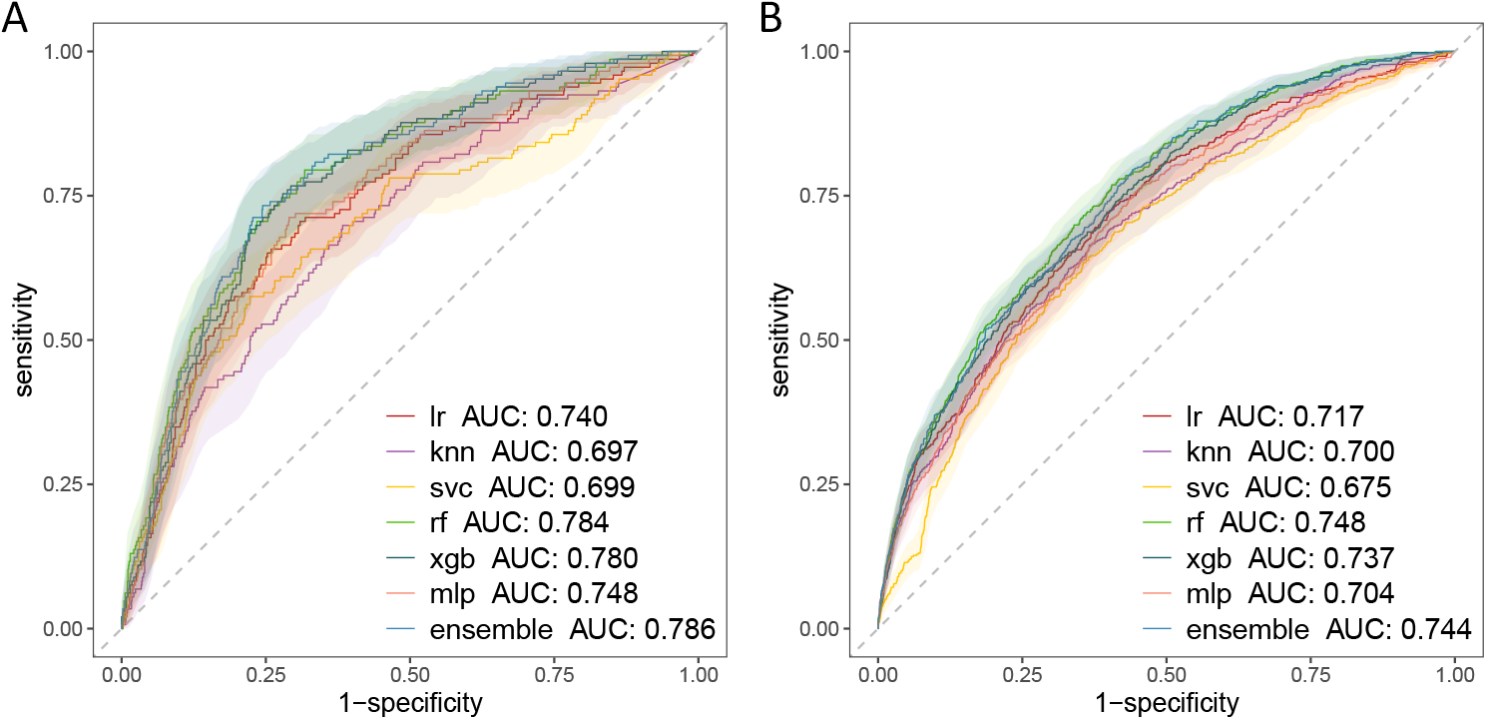
Receiver operating characteristic curves for the temporal validation of each model (**A**: PLAGH-ICU; **B**: MIMIC-IV). *lr* Logistic regression, *knn* K-Nearest Neighbor, *svc* Support Vector Classifier, *rf* Random Forest, *xgb* XGBoost eXtreme Gradient Boosting, *mlp* Multilayer Perceptron

The PLAGH-ICU-based ensemble models demonstrated optimal performance in the temporal validation set, recording an AUROC of 0.786 [0.748, 0.825]. In the MIMIC-IV models, the ensemble model achieved an AUROC of 0.744 [0.723, 0.766]. ROC curves for these models are presented in Fig. 1. In terms of AUROC, Random Forest, XGBoost, and Ensemble methods outperformed other algorithms.

**Fig. 2:**
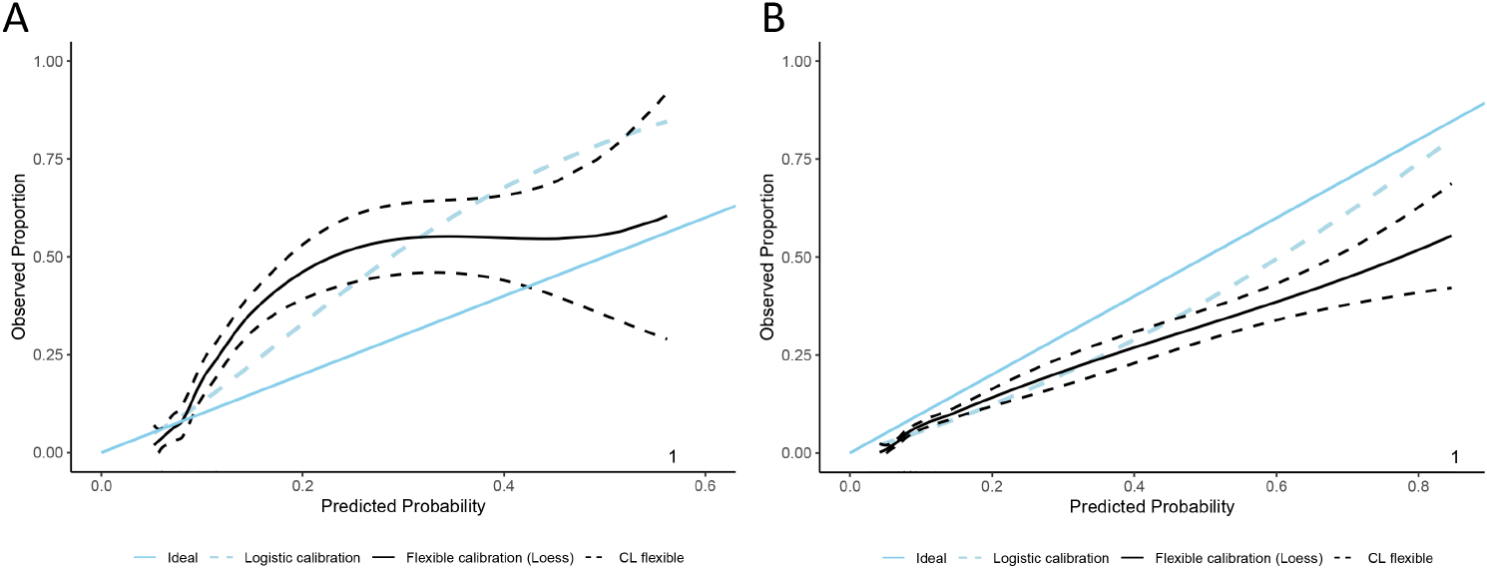
Probability calibration curves of ensemble models during temporal validation **(A**: PLAGH-ICU; **B**: MIMIC-IV)

Decision curve analysis, depicted in eFigure 3, revealed that in both datasets, the ensemble model outperformed others in lower high-risk threshold ranges, offering higher standardized net benefits. Calibration analysis of ensemble models developed from both datasets was conducted, focusing on Brier scores and the calibration curve metrics (Fig. 2). The PLAGH-ICU model recorded a Brier score of 0.1023 [0.0880, 0.1165], reflecting a higher predictive error, with its calibration curve intercept at 0.3308 [0.1519, 0.5096] and slope at 1.4766 [1.1950, 1.7582], showing significant calibration deviation. In contrast, the MIMIC-IV model, with a Brier score of 0.0554 [0.0517, 0.0592], demonstrated lower error. Its calibration curve featured an intercept of -0.6217 [-0.7160, -0.5274] and a slope of 1.085 [0.9755, 1.1947], indicating a smaller deviation from ideal calibration compared to the PLAGH-ICU model. In sensitivity analysis, the model’s performance remained stable in the temporal validation set after removing cases with missing data (eFigure 4).

The external validation involved assessing the PLAGH-ICU model on the MIMIC-IV dataset and vice versa. This process resulted in a reduction in model performance for both datasets. The PLAGH-ICU model reached a peak AUROC of 0.638 [0.628, 0.648] on MIMIC-IV, and the MIMIC-IV model attained an AUROC of 0.615 [0.585, 0.646] on PLAGH-ICU. The ROC curves of the external validation model are shown in eFigure 5.

### Interpretability

**Fig. 3:**
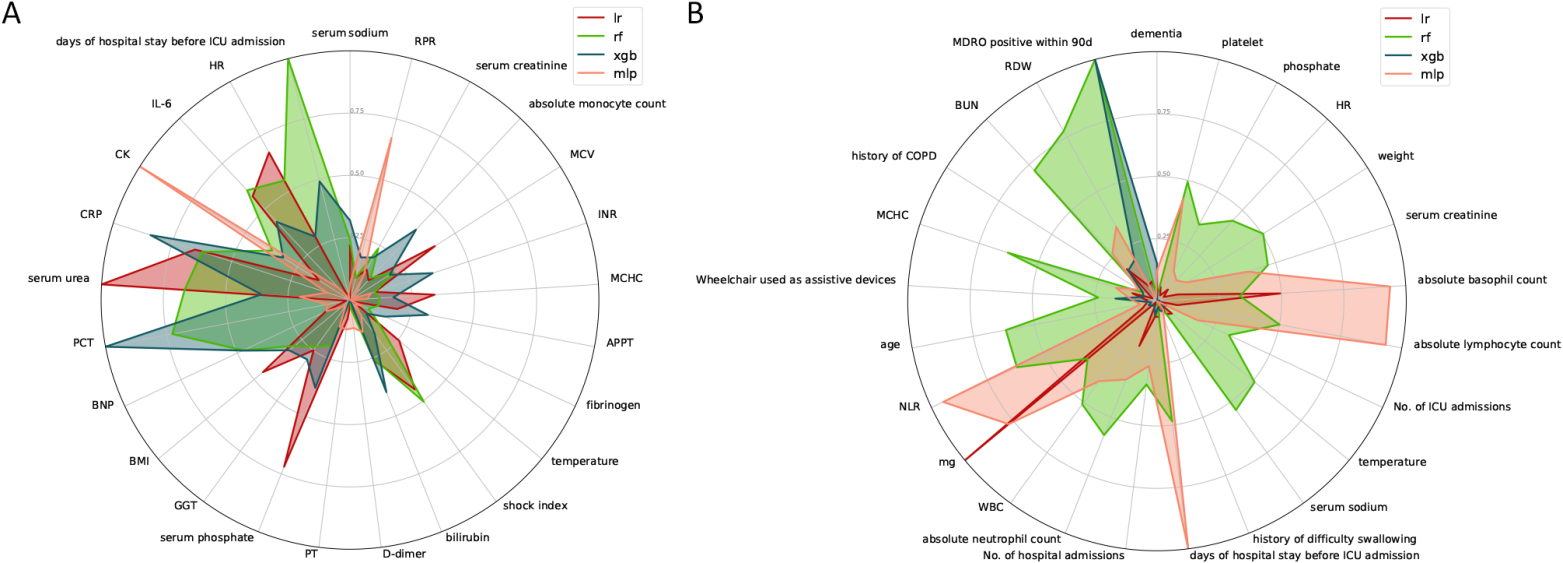
Radar Chart of Feature Importance Rankings for Each Model (**A**: PLAGH-ICU; **B**: MIMIC-IV). *lr* Logistic regression, *rf* Random Forest, *xgb* XGBoost eXtreme Gradient Boosting, *mlp* Multilayer Perceptron

**Fig. 4:**
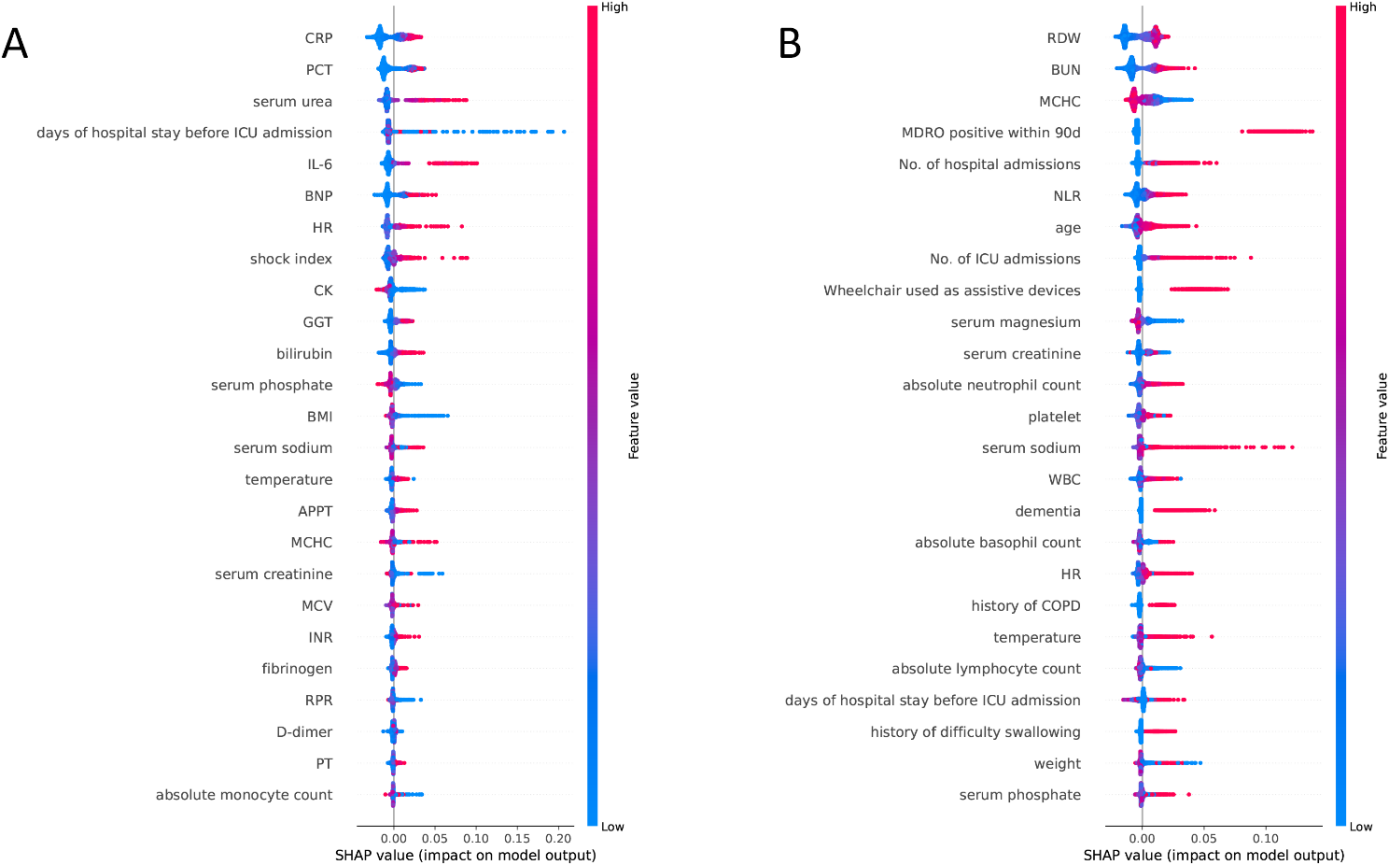
SHAP analysis (A: PLAGH-ICU; B: MIMIC-IV)

Feature importance in LR, RF, XGBoost, and MLP models is visually represented in radar charts (Fig. 3), highlighting notable differences in feature prioritization among these models. SHAP analysis (Fig. 4) elucidates the impact of individual variables on the random forest models. In the PLAGH-ICU context, biochemical markers like C-reactive protein (CRP), procalcitonin (PCT), serum urea, duration of pre-ICU hospital stay, and interleukin-6 (IL-6) emerged as highly influential, as indicated by their elevated SHAP values. The brain natriuretic peptide (BNP) also emerged as a significant predictor. In contrast, the MIMIC-IV model accentuated the importance of red cell distribution width (RDW), blood urea nitrogen (BUN), mean corpuscular hemoglobin concentration (MCHC), and MDRO positivity within 90 days. Elevated RDW and BUN levels, coupled with reduced MCHC, potentially signal an increased risk of MDRO carriage or infection. To further illustrate the interpretability of the model, a SHAP force plot analyzed the impact of features on the outcome for four patients (eFigure 6). In external validation, the SHAP analysis results, as shown in eFigure 7, displayed the top 15 features for early prediction of MDRO.

## Discussion

In this research, predictive models for early ICU MDRO colonization or infection were formulated utilizing the most significant 25 features from the PLAGH-ICU and MIMIC-IV datasets. These models reached AUROC of 0.786 and 0.744 in temporal validation, aligning with the acceptable accuracy standard cited in [31]. Excluding data that necessitate clinical input or are not routinely collected, these models are easily adaptable to hospital Electronic Health Record (EHR) systems. By analyzing the SHAP values, it is possible to identify the features that have the greatest impact on the predicted outcome, and also to reveal the complex nonlinear relationships between these features and the predicted outcomes. This interpretive analysis not only enhances the transparency of the model, but also provides valuable insights for clinical decision making. Clinicians can incorporate this information into their decision-making process to more accurately identify high-risk patients and adjust management strategies accordingly. In addition, individual patient SHAP analysis demonstrates how each characteristic affects their individual predicted outcomes. This personalized interpretation can help clinicians understand a specific patient’s unique risk profile, leading to a tailored treatment plan. Their application could assist clinicians in swiftly determining MDRO colonization or infection in new ICU patients, likely enhancing empirical antimicrobial usage and mitigating MDRO proliferation. Future implementations will focus on deploying these models in clinical settings with real-time data integration, facilitated by developing interfaces compatible with clinical EHR systems. The models will be incorporated into a clinical decision support system (CDSS) to deliver timely alerts and recommendations. The implementation will address challenges such as data access, privacy concerns, hardware and software integration, physician adoption, and regulatory compliance by engaging key stakeholders including clinicians, technology developers, and regulatory bodies. Additionally, a monitoring framework will be established to continuously assess and enhance the model’s performance in clinical environments, ensuring that it remains effective and relevant.

In the temporal validation, the performance of the RF, XGBoost and ensemble models, as measured by AUROC, surpassed that of LR, KNN, SVC, and MLP. This superiority may be attributed to the fact that RF, XGBoost, and ensemble models are all ensemble methods, which have certain advantages in handling complex data. By integrating the predictive outcome of multiple models, these methods enhance the stability and accuracy of the model [23, 32, 33]. Models derived from PLAGH-ICU and MIMIC-IV data showcase varied feature preferences. In the PLAGH-ICU models, the focus is on laboratory values and vital signs, with CRP, PCT, and IL-6 as primary indicators. Conversely, MIMIC-IV models prioritize pre-ICU information, including hospitalization count and recent MDRO detection, along with lab values like RDW, BUN, and MCHC. The variation in data completeness, particularly the higher absence of certain PLAGH-ICU indicators in the MIMIC-IV dataset, led to their exclusion in modeling. This limitation prevented evaluating these indicators’ effectiveness across both databases. The noticeable drop in model performance during external validation, attributed to differences in pathogen epidemiology and medical practices, underscores the potential benefit of developing unit-specific MDRO early warning models. Similar issues have been observed in other studies [34]. These factors underscore the significant challenges in creating predictive models with strong generalizability that can be applied across different institutions. Nevertheless, this remains a worthwhile endeavor. Collecting data from various hospitals and regions to construct universal predictive factors could potentially enhance the generalizability of these models.

This study employed only the initial EHR data from ICU admissions for model construction, confronting the inherent challenge of limited feature-target correlations, a common hurdle in MDRO prediction models typically struggling to attain high accuracy. Earlier research identifies key risk factors for MDRO infection, including age, immunodeficiency, invasive procedures, recent antibiotic use, repeated or prolonged hospitalizations, and prior MDRO colonization or infection [35, 36]. Yi Li et al. created a prediction model for carbapenem-resistant Klebsiella pneumoniae infection using data from three central Chinese hospitals’ ICUs, validated on three other hospitals’ data. They identified prior year colonization or infection, a CD4/CD8 ratio below 1, and over 48 h of parenteral nutrition as independent risk factors, achieving an AUROC of 0.844 in external validation [37]. Li Wang et al. conducted a retrospective analysis of 336 ICU patients from the First Affiliated Hospital of Xiamen University, identifying increased Pitt bacteremia scores (PBS), male gender, and elevated CRP levels as independent risk factors in their logistic regression model, with an external validation AUROC of 0.77 [38]. Wang et al. employed data from 688 ICU patients, utilizing Lasso and stepwise regression to extract nine independent MDRO infection risk factors for a backpropagation neural network (BPNN) model, validated externally with an AUC of 0.811 [39]. Jiang et al. analyzed data from 297 neuro ICU patients, finding tracheal intubation, arterial blood pressure monitoring, fever, antibiotic use, and pneumonia as independent MDRO infection risk factors through binary logistic regression [40]. While these studies highlight relevant risk factors, implementing these models directly in hospitals poses challenges. This difficulty is partly due to the lack of published code and model parameters in many studies, as well as the inability to directly apply models developed elsewhere to local hospital settings. This issue is exemplified by the significant performance drop observed when models developed in databases from different countries were validated against each other. Compared to previous studies, this study utilized multicenter data to establish a predictive model, featuring a larger volume of dataset and a more comprehensive set of features. This approach allows for the analysis of MDRO prediction models in various research contexts and provides valuable references for constructing models suitable for different institutions.

In aligning the predictive modeling with real-world clinical scenarios, this study’s approach extends beyond merely identifying MDRO infection, encompassing both MDRO colonization and infection in the positive group and including non-MDRO positive cultures and negative cultures in the negative group. This strategy likely accounts for the notable difference in feature selection compared to other studies. Under these parameters, traditional lab markers for infection might have reduced predictive effectiveness, while metrics indicative of immune compromise or systemic weakness might emerge as more predictive. In the MIMIC-IV dataset, the duration of antimicrobial usage, though considered, did not feature prominently, likely reflecting the short median pre-ICU hospitalization duration (0.1 day). Elevated BUN and creatinine levels, often associated with renal function and nutritional status [41], appeared to increase the likelihood of MDRO positivity in the model. This could be attributed to renal impairment in severe infections or underlying renal conditions leading to malnutrition or weakened immunity. SHAP analysis also suggests that lower BUN and creatinine levels correlate with higher MDRO positivity, potentially indicating compromised nutritional and immune status, as evidenced by diminished muscle metabolism (reflected in low creatinine levels), hindering the clearance of MDRO.

The models indicate that elevated liver function markers, specifically gamma-glutamyl transferase (GGT) and bilirubin, heighten the likelihood of MDRO positivity, likely due to their impact on immune and nutritional status [42, 43]. RDW, a measure of red blood cell size variability, traditionally linked to anemia [44], emerges as a significant predictor in our model. Elevated RDW levels can reflect a state of inflammation, where erythropoietin-driven erythropoiesis maintains hemoglobin levels until anemia eventually occurs. This fluctuation in red blood cell production causes size variations, hence the implication of RDW as an inflammation marker in our study. Moreover, the correlation between high RDW levels and nutritional deficiencies may further substantiate its predictive efficacy [45]. While elevated white blood cell count (WBC), CRP, and PCT are conventional bacterial infection indicators, the model’s varied reliance on these markers-particularly the underutilization of WBC in PLAGH-ICU and its lesser emphasis in MIMIC-IV-underscores their variable nature influenced by factors like age, immune status, and medication [46]. Notably, in PLAGH-ICU, shorter pre-ICU hospital stays surprisingly correlated with increased MDRO risk, possibly reflecting a higher likelihood of resistant bacteria carriage among patients transferred from other hospitals after prolonged treatment. This observation could also be linked to community-acquired MDRO prevalence, necessitating further research.

This study has several limitations. First, it is a retrospective study using electronic health record data, and prospective validation of the model is needed to truly assess its impact on improving clinical practice. Second, although the model developed using PLAGH-ICU data has acceptable classification capabilities, its precision, specifically the positive predictive value, is not high, particularly at probability thresholds ensuring higher recall rates, which might entail high costs if applied clinically at this stage. Third, as previously mentioned, models built using data from specific institutions might not be directly applicable in other medical facilities; incorporating data from different units could be necessary to develop models with strong generalizability. Additionally, although we examined the feature importance across different models, it only indicates the correlation between variables and model predictions, not causality, and caution is needed when interpreting and applying these features.

## Conclusion

Employing machine learning algorithms, this study developed models for predicting MDRO colonization or infection with data from MIMIC-IV and PLAGH-ICU. These models are instrumental in early identification of patients at high risk of MDRO colonization or infection upon ICU admission, a crucial step in managing antibiotic resistance and optimizing antimicrobial therapy. The models trained on ICU data from diverse geographic regions showed significant variance in feature selection and performance. This underscores the practicality of medical institutions using their own data to train models while integrating insights from broader research. Future endeavors should concentrate on refining the predictive efficacy of MDRO models and assessing their real-world applicability.

### Electronic supplementary material

Below is the link to the electronic supplementary material.

Supplementary Material 1

## Data Availability

The MIMIC-IV data are available on the website at https://physionet.org/content/mimiciv/2.2/. The other data in this article are available from the corresponding author on reasonable requests. The code for data processing, developing machine learning models, and performing statistical analysis can be obtained from GitHub (https://github.com/Brandon96-lab/MDRO_predict).

## Abbreviations

MDRO: Multidrug-Resistant Organisms
ICU: Intensive Care Units
ML: Machine Learning
PLAGH-ICU: People’s Liberation Army General Hospital
MIMIC-IV: Medical Information Mart for Intensive Care-IV
AUROC: Area Under the Receiver Operating Characteristics Curve
COVID-19: Coronavirus Disease 2019
LR: Logistic Regression
KNN: K-Nearest Neighbor
SVC: Support Vector Classifier
RF: Random Forest
XGBoost: eXtreme Gradient Boosting
MLP: Multilayer Perceptron
SHAP: Shapley Additive Explanations
BMI: Body Mass Index
CRP: C-reactive Protein
PCT: Procalcitonin
IL-6: Interleukin-6
BNP: Brain Natriuretic Peptide
RDW: Red Cell Distribution Width
BUN: Blood Urea Nitrogen
MCHC: Mean Corpuscular Hemoglobin Concentration
EHR: Electronic Health Record
GGT: Gamma-Glutamyl Transferase
