# Supplementary material for: Development and validation of machine learning models to predict MDRO colonization or infection on ICU admission by using electronic health record data
Source: Antimicrob Resist Infect Control. 2024 Jul 6;13:74. doi: 10.1186/s13756-024-01428-y (PMC11227715; doi:10.1186/s13756-024-01428-y)
Supplement: Supplementary file 1 — Supplementary Material 1 [file 13756_2024_1428_MOESM1_ESM.docx]

Appendix

Title: Development and Validation of Machine Learning Models to Predict MDRO Colonization or Infection on ICU admission by Using Electronic Health Record Data

Supplementary Tables

eTable 1. Baseline characteristics of vital signs and laboratory tests in PLAGH-ICU patients

eTable 2. Supplementary baseline characteristics of MIMIC-IV patients

eTable 3. Comparative table of available variables in PLAGH-ICU and MIMIC-IV data

eTable 4. Performance metrics of the ensemble models for MDRO predicting at various probability thresholds

Supplementary Figures

eFigure 1. Flowchart of patients inclusion and exclusion

eFigure 2. Quantity of major bacterial species and multi-drug resistant organisms (A: PLAGH-ICU；B: MIMIC-IV)

eFigure 3. Decision curve analysis for the temporal validation of each model (A: PLAGH-ICU; B: MIMIC-IV)

eFigure 4.The ROC curves for the sensitivity analysis (A: PLAGH-ICU; B: MIMIC-IV)

eFigure 5. Receiver operating characteristic curves for the external validation of each model (A: Assessing the PLAGH-ICU model on the MIMIC-IV dataset; B: Assessing the MIMIC-IV model on the PLAGH-ICU dataset)

eFigure 6. SHAP force plot for four selected patients. (A, B: PLAGH-ICU; C, D: MIMIC-IV)

eFigure 7. SHAP analysis in external validation(A: Assessing the PLAGH-ICU model on the MIMIC-IV dataset; B: Assessing the MIMIC-IV model on the PLAGH-ICU dataset)

eTable 1. Baseline characteristics of vital signs and laboratory tests in PLAGH-ICU patients

| Variable | Non-MDRO | MDRO | P-Value |
| --- | --- | --- | --- |
|  | (n = 3113) | (n = 423) |  |
| Vital Signs, Median [Q1, Q3] |  |  |  |
| Heart Rate (beats/min) | 79.0 [72.0,91.0] | 92.0 [78.8,112.0] | <0.001 |
| Systolic Blood Pressure (mmHg) | 126.0 [113.0,140.0] | 124.0 [110.0,140.0] | 0.040 |
| Diastolic Blood Pressure (mmHg) | 75.0 [66.0,83.0] | 72.0 [62.0,83.0] | 0.001 |
| Respiratory Rate (breaths/min) | 18.0 [18.0,18.5] | 18.0 [18.0,20.0] | <0.001 |
| Temperature (°C) | 36.6 [36.3,37.0] | 36.9 [36.5,37.5] | <0.001 |
| SPO_2_, median [Q1,Q3] | 97.0 [95.0,98.0] | 97.0 [94.0,98.0] | 0.036 |
| Laboratory Test Values, Median [Q1, Q3] |  |  |  |
| C-reactive Protein (mg/dL) | 1.6 [0.3,6.8] | 5.9 [2.2,14.1] | <0.001 |
| Procalcitonin (ng/mL) | 0.2 [0.1,1.2] | 1.3 [0.2,6.9] | <0.001 |
| Interleukin-6 (pg/mL) | 94.0 [36.2,262.2] | 180.9 [62.4,786.9] | <0.001 |
| White Blood Cell Count (×10^9/L) | 11.0 [8.0,14.9] | 10.9 [7.2,15.7] | 0.432 |
| Neutrophil Absolute Count | 9.6 [6.8,13.2] | 9.1 [6.1,13.8] | 0.205 |
| Basophil Absolute Count | 0.0 [0.0,0.0] | 0.0 [0.0,0.0] | 0.117 |
| Eosinophil Absolute Count | 0.0 [0.0,0.0] | 0.0 [0.0,0.1] | <0.001 |
| Monocyte Absolute Count | 0.4 [0.2,0.6] | 0.4 [0.2,0.7] | 0.236 |
| Lymphocyte Absolute Count | 0.8 [0.5,1.2] | 0.8 [0.5,1.2] | 0.221 |
| Hemoglobin Concentration (g/L) | 109.0 [93.0,126.0] | 99.0 [84.0,115.0] | <0.001 |
| Red Blood Cell Count (×10^12/L) | 3.7 [3.2,4.2] | 3.3 [2.9,3.9] | <0.001 |
| Red Blood Cell Distribution Width (%) | 13.5 [12.7,14.7] | 14.1 [13.1,15.7] | <0.001 |
| Mean Corpuscular Hemoglobin (pg) | 30.2 [29.0,31.4] | 30.3 [29.3,31.3] | 0.471 |
| Mean Corpuscular Hemoglobin Concentration (g/L) | 338.0 [329.0,347.0] | 336.0 [325.0,346.0] | 0.010 |
| Mean Corpuscular Volume (fL) | 88.9 [85.5,92.3] | 89.8 [86.0,93.5] | 0.002 |
| Platelet Count (×10^9/L) | 191.0 [139.0,250.0] | 185.0 [112.0,262.0] | 0.099 |
| Brain Natriuretic Peptide (U/L) | 192.4 [71.1,707.3] | 633.5 [173.9,2580.5] | <0.001 |
| Creatine Kinase (U/L) | 120.2 [62.1,273.8] | 75.6 [36.1,191.2] | <0.001 |
| Creatine Kinase-MB Isoenzyme (U/L) | 2.2 [1.3,4.3] | 1.9 [1.1,3.7] | 0.002 |
| Alkaline Phosphatase (U/L) | 60.6 [46.4,83.1] | 71.4 [50.7,108.5] | <0.001 |
| Alanine Aminotransferase (U/L) | 19.2 [11.5,38.3] | 20.5 [11.8,40.9] | 0.676 |
| Aspartate Aminotransferase (U/L) | 24.2 [16.1,45.2] | 23.4 [15.9,45.0] | 0.367 |
| Gamma-Glutamyl Transferase (U/L) | 23.8 [14.0,50.3] | 38.4 [18.1,93.8] | <0.001 |
| Albumin (g/L) | 30.9 [26.4,34.9] | 30.0 [26.5,34.0] | 0.075 |
| Serum Bilirubin (μmol/L) | 12.4 [8.5,19.3] | 16.2 [9.6,29.1] | <0.001 |
| Direct Bilirubin (μmol/L) | 5.0 [3.2,8.5] | 7.4 [4.4,15.6] | <0.001 |
| Activated Partial Thromboplastin Time (s) | 39.5 [35.5,44.5] | 42.2 [37.8,49.9] | <0.001 |
| International Normalized Ratio | 1.2 [1.1,1.4] | 1.3 [1.2,1.5] | <0.001 |
| D-dimer (μg/mL) | 3.1 [1.6,6.2] | 3.8 [2.4,7.4] | <0.001 |
| Prothrombin Time (s) | 15.3 [14.4,16.8] | 16.0 [14.7,17.9] | <0.001 |
| Partial Thromboplastin Time (s) | 15.2 [14.3,16.3] | 15.0 [14.0,16.3] | 0.051 |
| Fibrinogen (g/L) | 3.3 [2.4,4.5] | 3.8 [2.6,5.2] | <0.001 |
| Blood Creatinine (μmol/L) | 70.4 [55.2,93.9] | 73.7 [54.4,117.0] | 0.063 |
| Blood Urea Nitrogen (mmol/L) | 5.4 [4.0,7.8] | 7.3 [4.7,13.3] | <0.001 |
| Blood Potassium (mmol/L) | 4.0 [3.7,4.4] | 4.0 [3.6,4.4] | 0.397 |
| Blood Magnesium (mmol/L) | 0.8 [0.7,0.9] | 0.8 [0.7,0.9] | 0.001 |
| Blood Sodium (mmol/L) | 139.6 [136.6,142.4] | 138.4 [134.2,142.5] | 0.001 |
| Blood Calcium (mmol/L) | 2.0 [1.9,2.1] | 2.0 [1.9,2.1] | 0.562 |
| Blood Chloride (mmol/L) | 105.1 [101.6,108.3] | 103.8 [99.5,108.4] | 0.002 |
| Blood Glucose (mmol/L) | 8.2 [6.7,10.5] | 8.0 [6.4,10.6] | 0.103 |
| Plasminogen Activator (PA) | 74.0 [63.0,83.0] | 68.0 [56.5,79.0] | <0.001 |
| Hematocrit Packed Cell Volume (fL) | 0.3 [0.3,0.4] | 0.3 [0.3,0.4] | <0.001 |
| Mean Platelet Volume | 10.4 [9.6,11.1] | 10.7 [9.8,11.7] | <0.001 |
| Serum Phosphate (mmol/L) | 1.2 [0.9,1.4] | 1.0 [0.8,1.3] | <0.001 |
| Reactive Protein Ratio (RPR) | 0.1 [0.1,0.1] | 0.1 [0.1,0.1] | 0.003 |
| Neutrophil to Lymphocyte Ratio (NLR) | 12.4 [7.3,20.0] | 11.8 [7.5,20.5] | 0.858 |

| eTable 2. Supplementary baseline characteristics of MIMIC-IV patientsVariable | Non-MDRO | MDRO | *P*-Value |
| --- | --- | --- | --- |
|  | (n = 28863) | (n = 2788) |  |
| race, n (%) |  |  | <0.001 |
| Asian | 895 (3.1) | 88 (3.2) |  |
| Black/African American | 3230 (11.2) | 312 (11.2) |  |
| Hispanic/Latino | 1232 (4.3) | 103 (3.7) |  |
| Other | 4198 (14.5) | 323 (11.6) |  |
| White | 19308 (66.9) | 1962 (70.4) |  |
| ICU type, n (%) |  |  | <0.001 |
| Cardiac Vascular Intensive Care Unit (CVICU) | 3888 (13.5) | 112 (4.0) |  |
| Coronary Care Unit (CCU) | 3186 (11.0) | 221 (7.9) |  |
| Medical Intensive Care Unit (MICU) | 7970 (27.6) | 1098 (39.4) |  |
| Medical/Surgical Intensive Care Unit (MICU/SICU) | 5869 (20.3) | 749 (26.9) |  |
| Surgical Intensive Care Unit (SICU) | 3786 (13.1) | 345 (12.4) |  |
| Trauma SICU (TSICU) | 2552 (8.8) | 231 (8.3) |  |
| Other | 1612 (5.7) | 32 (1.1) |  |
| History, n (%) |  |  |  |
| Unintentional Weight Loss > 10 lbs | 1922 (6.7) | 225 (8.1) | 0.005 |
| Use of Assistive Devices: Cane | 1989 (6.9) | 169 (6.1) | 0.105 |
| Use of Assistive Devices: Walker | 2221 (7.7) | 323 (11.6) | <0.001 |
| Use of Assistive Devices: Wheelchair | 1012 (3.5) | 277 (9.9) | <0.001 |
| chronic obstructive pulmonary disease | 2450 (8.5) | 448 (16.1) | <0.001 |
| Undergoing Dialysis | 982 (3.4) | 122 (4.4) | 0.009 |
| Difficulty Swallowing | 2053 (7.1) | 347 (12.4) | <0.001 |
| Intravenous Access | 12683 (43.9) | 1496 (53.7) | <0.001 |
| Anemia | 1547 (5.4) | 246 (8.8) | <0.001 |
| Alcohol Use | 1960 (6.8) | 168 (6.0) | 0.133 |
| Gastrointestinal Bleed | 1225 (4.2) | 167 (6.0) | <0.001 |
| Pancreatitis | 292 (1.0) | 35 (1.3) | 0.264 |
| Seizures | 697 (2.4) | 98 (3.5) | <0.001 |
| Angina | 786 (2.7) | 52 (1.9) | 0.008 |
| Hypertension | 10052 (34.8) | 1028 (36.9) | 0.032 |
| Cardiovascular Pacemaker | 863 (3.0) | 122 (4.4) | <0.001 |
| Treatment, n (%) |  |  |  |
| Days of Immunosuppressant Use Before ICU, median [Q1,Q3] | 0.0 [0.0,0.0] | 0.0 [0.0,0.0] | <0.001 |
| Days of Antimicrobial Use Before ICU, median [Q1,Q3] | 0.0 [0.0,0.0] | 0.0 [0.0,0.0] | 0.667 |
| Antimicrobial Use on First Day of Admission, n (%) | 18590 (64.4) | 2312 (82.9) | <0.001 |
| Immunosuppressant Use on First Day of Admission, n (%) | 3479 (12.1) | 431 (15.5) | <0.001 |
| Vasopressor Use Before ICU, n (%) | 953 (3.3) | 111 (4.0) | 0.065 |
| Vasopressor Use on First Day of ICU, n (%) | 9946 (34.5) | 1093 (39.2) | <0.001 |
| Surgical, n (%) | 9198 (31.9) | 585 (21.0) | <0.001 |
| Invasive Line Placement on First Day of ICU, n (%) | 15993 (55.4) | 1757 (63.0) | <0.001 |
| Glasgow Coma Scale (Median [Q1, Q3]) | 15.0 [15.0,15.0] | 15.0 [14.0,15.0] | <0.001 |
| Sequential Organ Failure Assessment (Median [Q1, Q3]) | 4.0 [2.0,7.0] | 5.0 [3.0,8.0] | <0.001 |
| Acute Physiology Score III (Median [Q1, Q3]) | 43.0 [32.0,56.0] | 52.0 [40.0,66.0] | <0.001 |
| Vital Signs, Median [Q1, Q3] |  |  |  |
| Heart Rate (beats/min) | 88.0 [77.0,104.0] | 92.0 [80.0,108.2] | <0.001 |
| Systolic Blood Pressure (mmHg) | 122.0 [107.0,140.0] | 119.0 [104.0,136.0] | <0.001 |
| Diastolic Blood Pressure (mmHg) | 67.0 [57.0,80.0] | 65.0 [54.0,77.0] | <0.001 |
| Respiratory Rate (breaths/min) | 19.0 [16.0,23.0] | 20.0 [16.0,24.0] | <0.001 |
| Temperature (°C) | 36.7 [36.4,37.1] | 36.8 [36.4,37.2] | <0.001 |
| SpO2 (%) | 98.0 [95.0,100.0] | 98.0 [95.0,100.0] | <0.001 |
| Laboratory Test Values, Median [Q1, Q3] |  |  |  |
| White Blood Cell Count (×10^9/L) | 10.8 [7.6,15.2] | 11.8 [7.9,17.1] | <0.001 |
| Absolute Neutrophil Count (×10^9/L) | 8.7 [5.6,12.7] | 9.7 [6.0,14.4] | <0.001 |
| Absolute Basophil Count (×10^9/L) | 0.0 [0.0,0.0] | 0.0 [0.0,0.0] | <0.001 |
| Absolute Eosinophil Count (×10^9/L) | 0.1 [0.0,0.1] | 0.0 [0.0,0.1] | <0.001 |
| Absolute Monocyte Count (×10^9/L) | 0.5 [0.3,0.8] | 0.5 [0.3,0.8] | 0.324 |
| Absolute Lymphocyte Count (×10^9/L) | 1.1 [0.7,1.8] | 1.0 [0.6,1.6] | <0.001 |
| Hemoglobin (g/dL) | 10.8 [9.1,12.6] | 10.2 [8.8,11.8] | <0.001 |
| Hematocrit (%) | 32.9 [27.9,38.2] | 31.8 [27.6,36.4] | <0.001 |
| Red Blood Cell Count (×10^12/L) | 3.6 [3.0,4.2] | 3.5 [3.0,4.0] | <0.001 |
| Red Cell Distribution Width (%) | 14.6 [13.4,16.3] | 15.6 [14.3,17.2] | <0.001 |
| Mean Corpuscular Hemoglobin (pg) | 30.2 [28.6,31.6] | 29.9 [28.2,31.3] | <0.001 |
| Mean Corpuscular Hemoglobin Concentration (g/dL) | 32.9 [31.7,34.0] | 32.3 [31.1,33.5] | <0.001 |
| Mean Corpuscular Volume (fL) | 91.0 [87.0,96.0] | 92.0 [87.0,96.0] | <0.001 |
| Platelet Count (×10^9/L) | 202.0 [142.0,276.0] | 219.0 [146.0,300.0] | <0.001 |
| Partial Thromboplastin Time (seconds) | 30.1 [26.6,35.8] | 31.5 [27.3,38.4] | <0.001 |
| Prothrombin Time (seconds) | 13.9 [12.2,16.6] | 14.5 [12.8,18.3] | <0.001 |
| International Normalized Ratio | 1.2 [1.1,1.5] | 1.3 [1.1,1.7] | <0.001 |
| Anion Gap (mEq/L) | 15.0 [13.0,18.0] | 15.0 [13.0,18.0] | <0.001 |
| Serum Potassium (mmol/L) | 4.2 [3.8,4.6] | 4.2 [3.7,4.7] | 0.084 |
| Blood Glucose (mg/dL) | 129.0 [105.0,168.0] | 128.0 [103.0,172.0] | 0.467 |
| Serum Creatinine (mg/dL) | 1.0 [0.7,1.5] | 1.2 [0.8,2.0] | <0.001 |
| Blood Urea Nitrogen (mg/dL) | 20.0 [14.0,34.0] | 28.0 [17.0,45.0] | <0.001 |
| Serum Sodium (mmol/L) | 138.0 [135.0,141.0] | 138.0 [135.0,142.0] | <0.001 |
| Serum Chloride (mmol/L) | 103.0 [99.0,107.0] | 103.0 [99.0,108.0] | 0.108 |
| Total Carbon Dioxide (mEq/L) | 25.0 [22.0,28.0] | 25.0 [21.0,30.0] | 0.994 |
| Base Excess (mEq/L) | 0.0 [-5.0,1.0] | -1.0 [-5.0,2.0] | 0.927 |
| Serum Bicarbonate (mEq/L) | 23.0 [20.0,26.0] | 23.0 [19.0,26.0] | 0.419 |
| Reactive Protein Ratio (RPR), Median [Q1, Q3] | 0.1 [0.1,0.1] | 0.1 [0.1,0.1] | 0.402 |
| Neutrophil to Lymphocyte Ratio (NLR), Median [Q1, Q3] | 7.5 [4.1,13.9] | 9.7 [5.1,18.6] | <0.001 |

**eTable 3** Comparative Table of Variable Completeness in PLAGH-ICU and MIMIC-IV Datasets

| Variable | PLAGH-ICU (%) | MIMIC-IV (%) |
| --- | --- | --- |
| Age | 100 | 100 |
| Gender | 100 | 100 |
| Weight | 95.16 | 98.03 |
| Height | 96.69 | 51.73 |
| BMI | 94.99 | 48.49 |
| Admission Type | 100 | 100 |
| race | - | 100 |
| ICU type | - | 100 |
| Number of Hospital Admissions | 100 | 100 |
| Number of ICU Admissions | 100 | 100 |
| Days in Hospital Before ICU Admission | 100 | 100 |
| MDRO Detected Within 90 Days Prior to ICU Admission | 100 | 100 |
| Comorbidities | 100 | 100 |
| History | 0 | 100 |
| Charlson Comorbidity Index | 100 | 100 |
| Glasgow Coma Scale | 0 | 99.86 |
| Sequential Organ Failure Assessment | 0 | 100 |
| Acute Physiology Score III | 0 | 100 |
| Shock Index | 99.97 | 99.59 |
| Vital Signs |  |  |
| Heart Rate | 100 | 99.86 |
| Systolic Blood Pressure | 99.97 | 99.61 |
| Diastolic Blood Pressure | 99.97 | 99.60 |
| Respiratory Rate | 100 | 99.58 |
| Temperature | 100 | 96.62 |
| SpO_2_ | 26.13 | 99.84 |
| Laboratory Test Values |  |  |
| White Blood Cell Count | 100 | 99.90 |
| Absolute Neutrophil Count | 99.94 | 73.23 |
| Absolute Basophil Count | 99.97 | 73.23 |
| Absolute Eosinophil Count | 99.97 | 73.23 |
| Absolute Monocyte Count | 99.94 | 73.23 |
| Absolute Lymphocyte Count | 99.94 | 73.30 |
| Hemoglobin | 100 | 99.89 |
| Hematocrit | 99.92 | 99.92 |
| Red Blood Cell Count | 100 | 99.89 |
| Red Cell Distribution Width | 99.92 | 99.87 |
| Mean Corpuscular Hemoglobin | 100 | 99.88 |
| Mean Corpuscular Hemoglobin Concentration | 100 | 99.89 |
| Mean Corpuscular Volume | 100 | 99.89 |
| Platelet Count | 100 | 99.90 |
| Activated Partial Thromboplastin Time | 99.97 | 0.00 |
| Partial Thromboplastin Time | 99.97 | 93.52 |
| Prothrombin Time | 99.97 | 93.93 |
| International Normalized Ratio | 99.97 | 93.94 |
| Anion Gap | 0.00 | 99.76 |
| Serum Potassium | 100 | 99.82 |
| Blood Glucose | 85.80 | 99.92 |
| Serum Creatinine | 99.92 | 99.91 |
| Blood Urea Nitrogen | 99.92 | 99.92 |
| Serum Sodium | 100 | 99.92 |
| Serum Chloride | 99.77 | 99.92 |
| Partial Pressure of Carbon Dioxide | 0.00 | 54.81 |
| Partial Pressure of Oxygen | 0.00 | 54.81 |
| Total Carbon Dioxide | 0.00 | 54.81 |
| Base Excess | 0.00 | 54.81 |
| Blood pH | 0.00 | 54.81 |
| Lactate | 0.00 | 35.26 |
| C-reactive Protein | 99.77 | 3.33 |
| Procalcitonin | 90.90 | 0.00 |
| Interleukin-6 | 82.47 | 0.00 |
| Brain Natriuretic Peptide | 96.23 | 13.56 |
| Creatine Kinase | 99.80 | 36.29 |
| Creatine Kinase-MB Isoenzyme | 98.93 | 33.52 |
| Alkaline Phosphatase | 99.32 | 63.25 |
| Alanine Aminotransferase | 100 | 63.48 |
| Aspartate Aminotransferase | 99.92 | 63.79 |
| Gamma-Glutamyl Transferase | 99.41 | 0.98 |
| Albumin | 100 | 52.21 |
| Serum Bilirubin | 100 | 63.10 |
| D-dimer | 99.92 | 0.30 |
| Fibrinogen | 99.97 | 29.10 |
| Blood Magnesium | 99.80 | 97.24 |
| Blood Calcium | 100 | 93.96 |
| Serum Bicarbonate | 0.00 | 99.84 |
| Plasminogen Activator | 99.97 | 0.00 |
| Direct Bilirubin | 99.97 | 6.34 |
| Hematocrit Packed Cell Volume | 100 | 0.00 |
| Mean Platelet Volume | 98.39 | 0.00 |
| Serum Phosphate | 99.89 | 94.02 |
| Reactive Protein Ratio | 99.92 | 99.95 |
| Neutrophil to Lymphocyte Ratio | 99.94 | 73.23 |

eTable 4. Performance metrics of the ensemble models for MDRO predicting at various probability thresholds

| Probability Threshold a | Count | Accuracy (%) | Sensitivity (%) | Specificity (%) | Positive Predictive Value (%) | Negative Predictive Value (%) | F1-score |
| --- | --- | --- | --- | --- | --- | --- | --- |
| PLAGH-ICU | | | | | | | |
| 0.054628 | 1094 | 17.25 | 99.32 | 5.29 | 13.25 | 98.15 | 0.233871 |
| 0.056274 | 997 | 25.35 | 97.95 | 14.77 | 14.34 | 98.01 | 0.250219 |
| 0.058482 | 871 | 35.63 | 95.21 | 26.95 | 15.96 | 97.47 | 0.273353 |
| 0.06197 | 761 | 44.51 | 92.47 | 37.52 | 17.74 | 97.16 | 0.297685 |
| 0.066032 | 668 | 51.22 | 86.99 | 46.01 | 19.01 | 96.04 | 0.312039 |
| 0.072589 | 544 | 61.15 | 83.56 | 57.88 | 22.43 | 96.03 | 0.353623 |
| 0.080705 | 437 | 69.08 | 78.08 | 67.76 | 26.09 | 95.5 | 0.391081 |
| 0.096343 | 328 | 76.48 | 69.86 | 77.45 | 31.1 | 94.63 | 0.43038 |
| 0.121064 | 218 | 81.18 | 50.68 | 85.63 | 33.94 | 92.26 | 0.406593 |
| 0.166302 | 110 | 85.02 | 28.77 | 93.21 | 38.18 | 89.98 | 0.328125 |
| MIMIC-IV | | | | | | | |
| 0.041733 | 8144 | 6.02 | 100 | 0.01 | 6 | 100 | 0.113286 |
| 0.047672 | 7786 | 10.36 | 99.59 | 4.66 | 6.25 | 99.44 | 0.117704 |
| 0.055064 | 6861 | 21.47 | 97.55 | 16.61 | 6.95 | 99.07 | 0.129796 |
| 0.063215 | 5839 | 33.58 | 93.87 | 29.73 | 7.86 | 98.7 | 0.14507 |
| 0.071587 | 4907 | 44.31 | 87.93 | 41.52 | 8.76 | 98.18 | 0.159377 |
| 0.081481 | 3887 | 55.87 | 79.96 | 54.34 | 10.06 | 97.7 | 0.178702 |
| 0.09379 | 2904 | 66.42 | 67.28 | 66.37 | 11.33 | 96.95 | 0.193929 |
| 0.111372 | 1944 | 76.69 | 54.6 | 78.1 | 13.73 | 96.42 | 0.219482 |
| 0.149455 | 953 | 86.74 | 37.01 | 89.92 | 18.99 | 95.72 | 0.25104 |

a Probability thresholds were established based on the incremental percentiles of the predicted risk.

Taking the PLAGH-ICU, which admits approximately 200 patients monthly, as an example, further analysis was conducted on the predictive functionality of the model used in this study. To ensure a detection rate of over 90% with a probability cutoff of 0.0620, the model predicted 133 individuals as positive, with 25 actual positive cases, potentially missing 2 cases; 67 were predicted negative, of which 66 were actually negative. For a detection rate of over 80% with a probability cutoff of 0.0726, the model predicted 95 individuals as positive, with 21 actual positive cases, potentially missing 4 cases; 105 were predicted negative, with 101 actually negative. At a detection rate of about 70%, with a probability threshold of 0.0963, the model predicted 57 individuals as positive, with 18 actual positive cases, potentially missing about 7 cases; 143 were predicted negative, with 135 actually negative. Based on these results, institutions can decide on cutoff values based on different costs and benefits. However, this study indicates that the model’s performance is limited, and the cost of identifying MDRO as comprehensively as possible remains high, necessitating ongoing optimization of model performance.


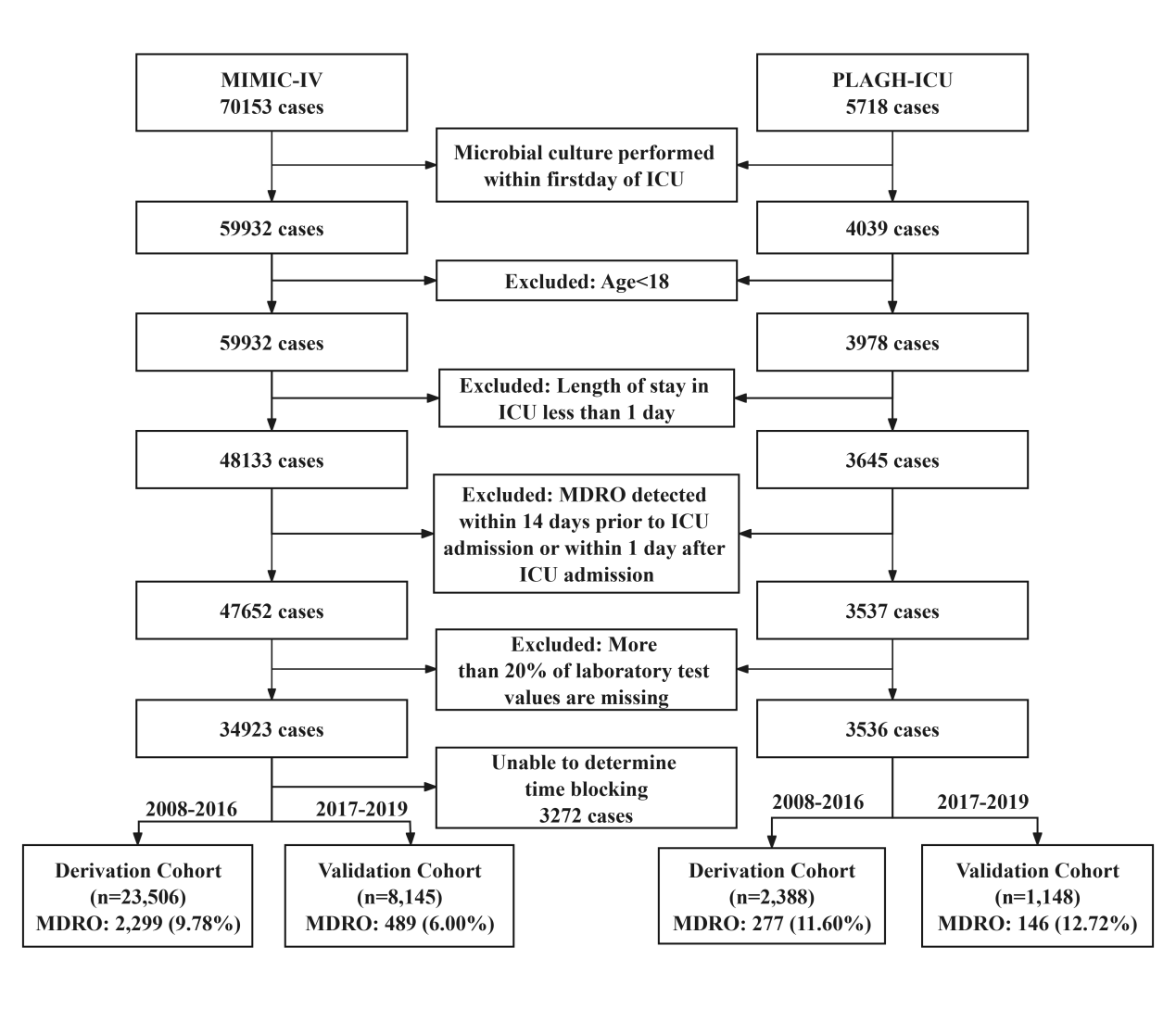


eFigure 1. Flowchart of patients inclusion and exclusion


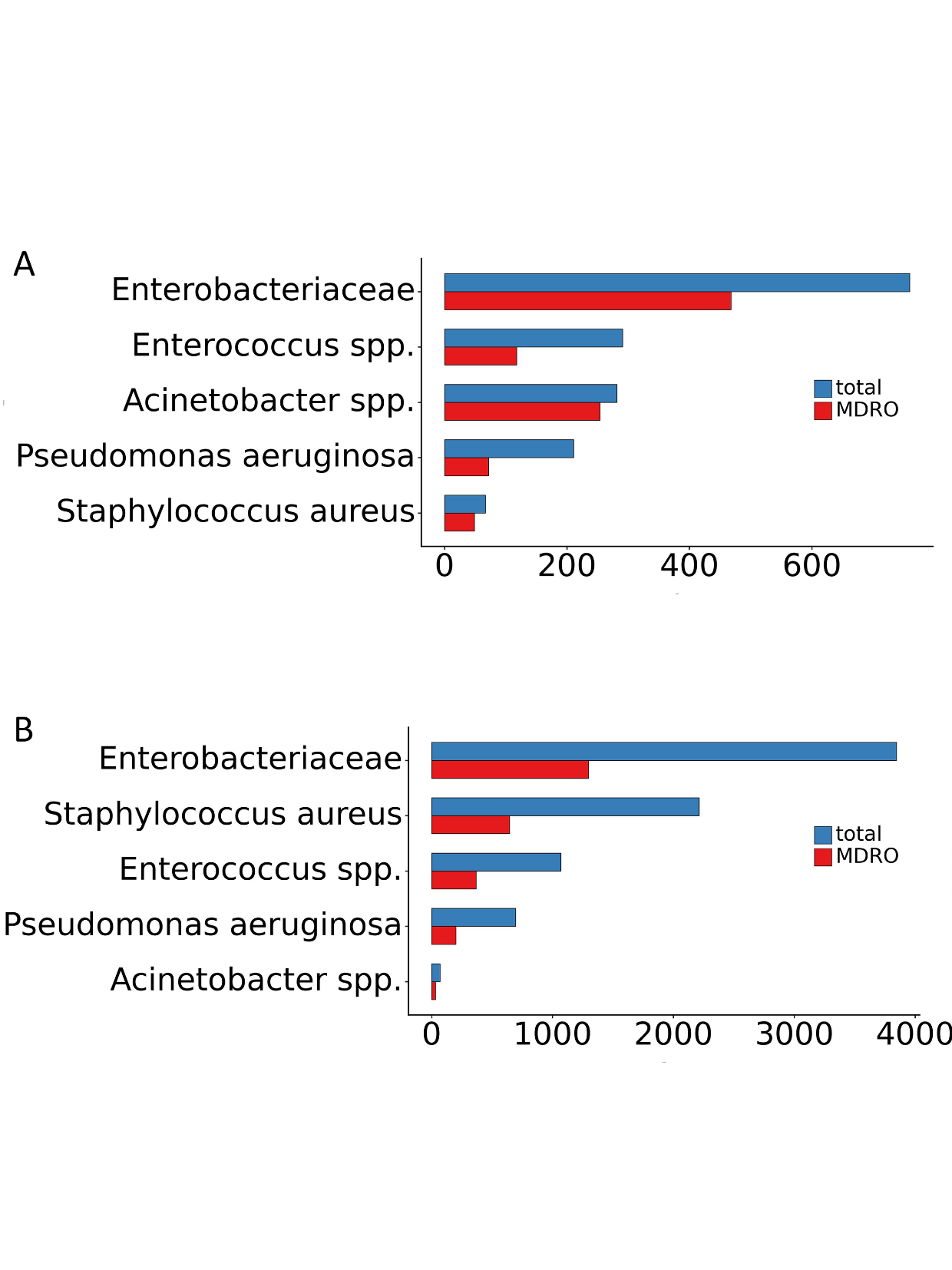


eFigure 2. Quantity of major bacterial species and multi-drug resistant organisms (A: PLAGH-ICU；B: MIMIC-IV)


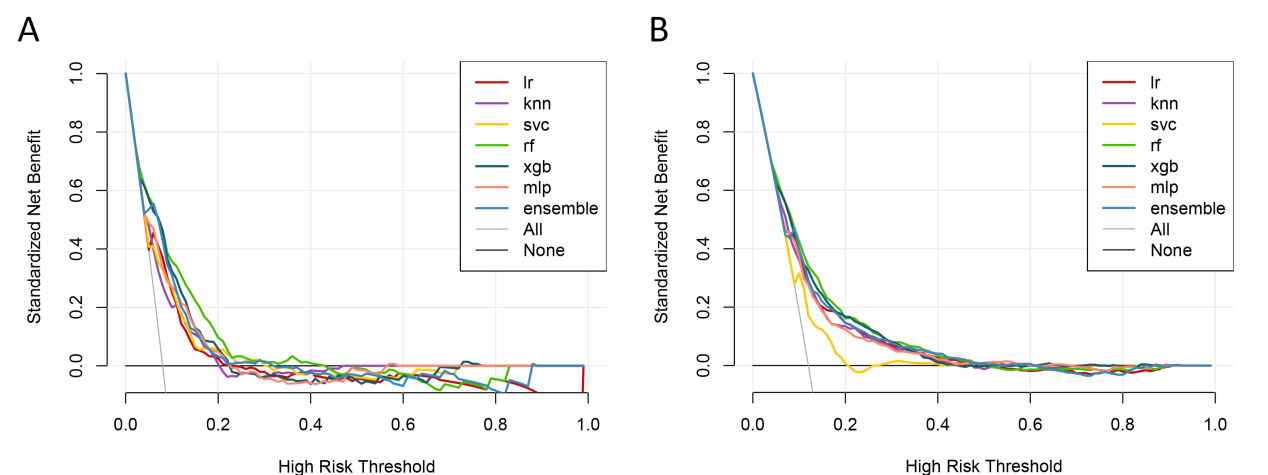


eFigure 3. Decision curve analysis for the temporal validation of each model (A: PLAGH-ICU; B: MIMIC-IV). *lr* Logistic regression, *knn* K-Nearest Neighbor, *svc* Support Vector Classifier, *rf* Random Forest, *xgb* XGBoost eXtreme Gradient Boosting, *mlp* Multilayer Perceptron


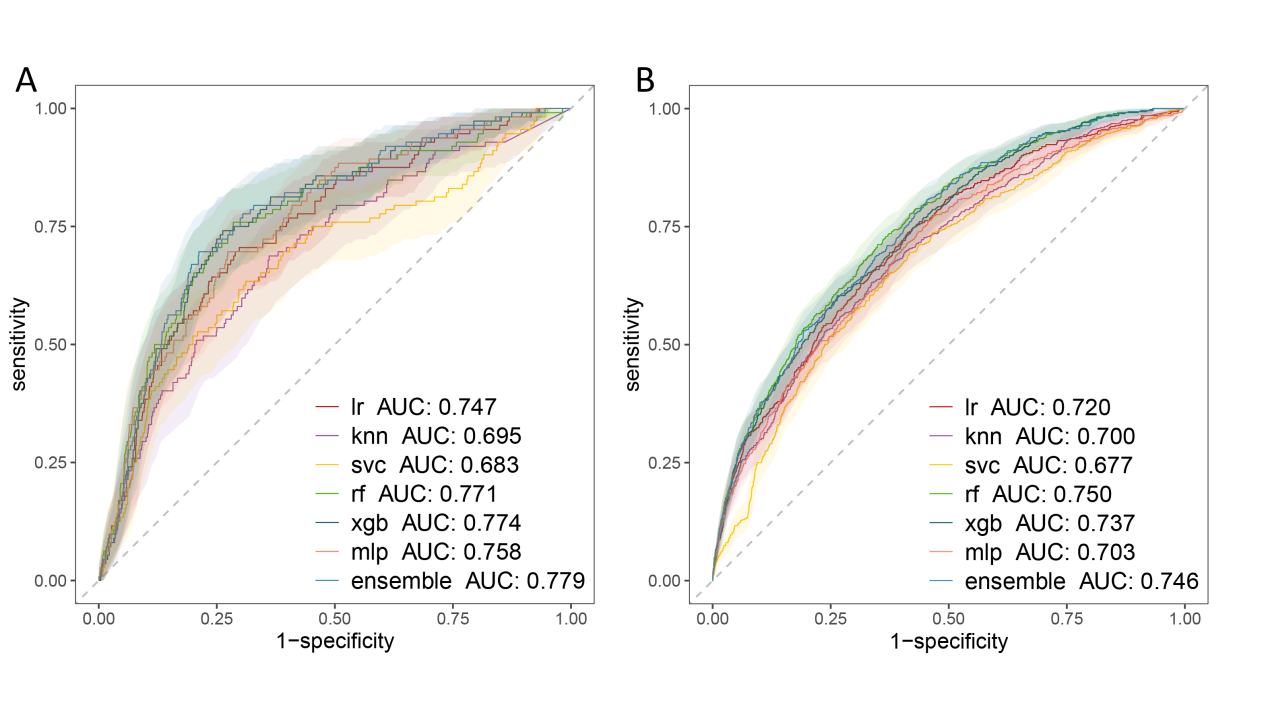


eFigure 4.The ROC curves for the sensitivity analysis (A: PLAGH-ICU; B: MIMIC-IV). *lr* Logistic regression, *knn* K-Nearest Neighbor, *svc* Support Vector Classifier, *rf* Random Forest, *xgb* XGBoost eXtreme Gradient Boosting, *mlp* Multilayer Perceptron


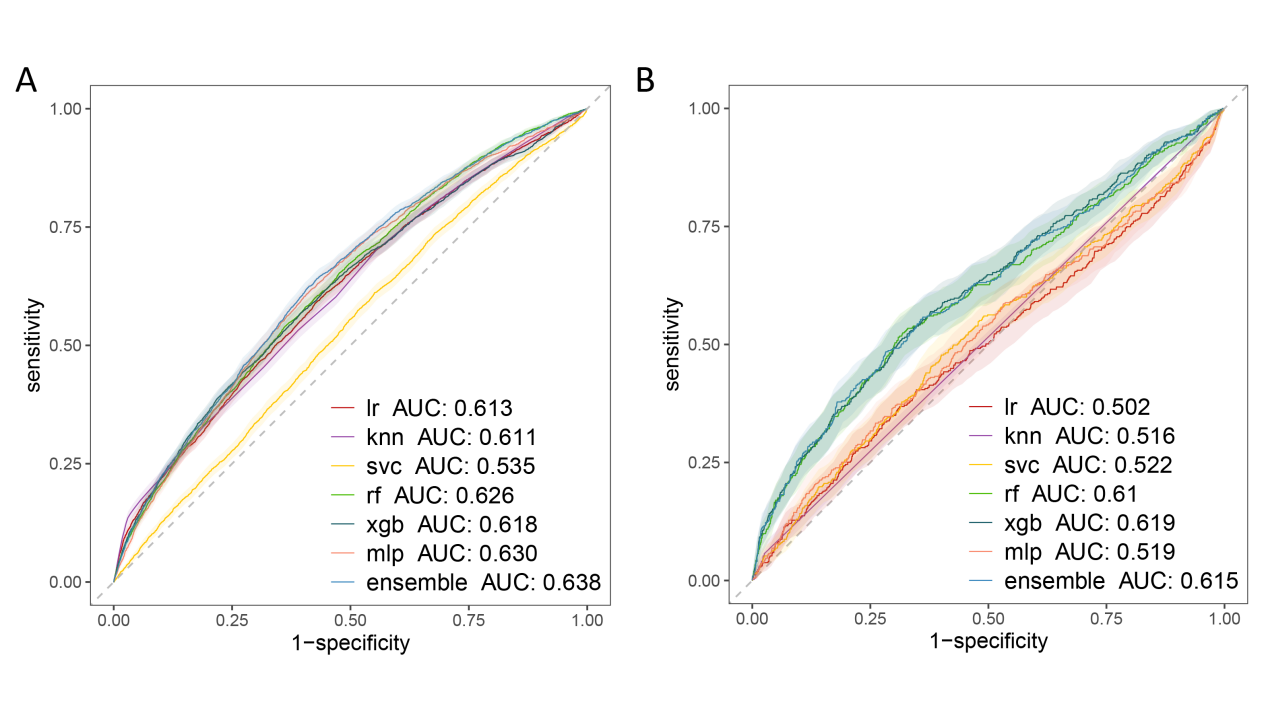


eFigure 5. Receiver operating characteristic curves for the external validation of each model (A: Assessing the PLAGH-ICU model on the MIMIC-IV dataset; B: Assessing the MIMIC-IV model on the PLAGH-ICU dataset). *lr* Logistic regression, *knn* K-Nearest Neighbor, *svc* Support Vector Classifier, *rf* Random Forest, *xgb* XGBoost eXtreme Gradient Boosting, *mlp* Multilayer Perceptron

To illustrate the interpretability of the model, eFigure 4 shows individual force plots for four patients regarding the occurrence of MDRO. eFigure 4A and B display the impact of features on outcomes for two cases from PLAGH-ICU. The model predicts a positive MDRO occurrence for one patient (eFigure 4A). In this case, the longest red bar corresponds to IL-6 (5000 pg/mL), which is the most significant predictor for MDRO occurrence. This is followed by PCT (2.5 ng/mL) and CRP (9.39 mg/dL). Similarly, a patient without MDRO is predicted as negative (eFigure 4B). The three variables with the most substantial negative impact are CRP (0.193 mg/dL), PCT (0.064 ng/mL), and heart rate (70 bpm). Additionally, eFigure 4C and D display the impact of features on outcomes for two cases from MIMIC-IV. The model predicts a positive MDRO occurrence for one patient (eFigure 4C). In this case, the top three positive influencers are BUN (116 mg/dL), MCHC (30.5 g/dL), and RDW (18%). Similarly, a patient without MDRO is predicted as negative (eFigure 4D). The three variables with the most substantial negative impact are RDW (13.2%), BUN (17 mg/dL), and MCHC (33.3 g/dL).

eFigure 6. SHAP force plot for four selected patients. (A, B: PLAGH-ICU; C, D: MIMIC-IV). A and B show patients with positive (MDRO) and negative (Non-MDRO) predictions in the PLAGH-ICU, respectively. C and D show patients with positive (MDRO) and negative (Non-MDRO) predictions in the MIMIC-IV, respectively.


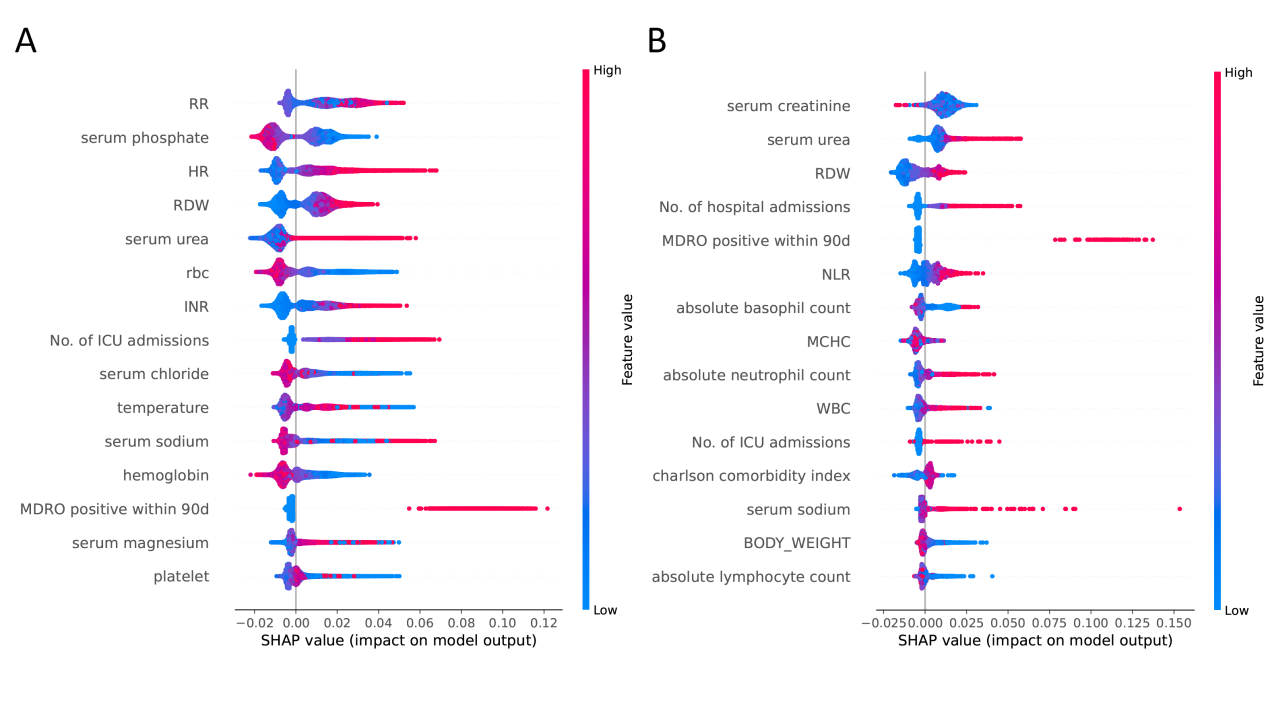


eFigure 7. SHAP analysis in external validation(A: Assessing the PLAGH-ICU model on the MIMIC-IV dataset; B: Assessing the MIMIC-IV model on the PLAGH-ICU dataset).
